# We’re not all cut from the same cloth: TAILORing treatments for children with chronic conditions

**DOI:** 10.1186/s41687-019-0117-2

**Published:** 2019-04-29

**Authors:** Rebecca N. Jerome, Jill M. Pulley, Terri L. Edwards, Alyssa B. Dickerson, Douglas Conway, Sara L. Van Driest, Gordon R. Bernard, Paul A. Harris

**Affiliations:** 1 Vanderbilt Institute for Clinical and Translational Research, 2525 West End Avenue, Suite 600, Nashville, Tennessee 37203 USA; 2 Departments of Pediatrics and Medicine, Vanderbilt University Medical Center, 2200 Children’s Way, 8232 Doctors’ Office Tower, Nashville, TN 37232 USA

**Keywords:** Personalized medicine, Autism spectrum disorders, Attention deficit hyperactivity disorder, N-of-1 studies, Patient reported outcomes, Patient engagement, Shared decision making

## Abstract

**Background:**

Finding the optimal treatment for a chronic condition can be a complex and lengthy endeavor for both the patient and the clinician. To address this challenge, we developed an “N-of-1” quality improvement infrastructure to aid providers and patients in personalized treatment decision-making using systematic assessment of patient-reported outcomes during routine care.

**Methods:**

Using the REDCap data management infrastructure, we implemented three pediatric pilots of the Treatment Assessments in the Individual Leading to Optimal Regimens (TAILOR) tool, including children receiving early intervention, children with attention deficit hyperactivity disorder, and children with challenging behaviors in the classroom setting. This retrospective review of data summarizes utilization and satisfaction data during our pilot experience with the tool.

**Results:**

The three pilots included a combined total of 109 children and 39 healthcare providers, with 67 parents and 77 teachers invited to share data using brief surveys administered using TAILOR. Overall survey response rates ranged from 38% to 84% across the three pilots, with response rates notably higher among teachers as compared with parents. Satisfaction data indicated positive impressions of the tool’s utility.

**Discussion:**

These experiences show the utility of the TAILOR framework for supporting collection and incorporation of patient-reported outcomes into the care of individuals with chronic conditions.

## Background

Chronic diseases are increasingly prevalent [1], and managing them can be a complex endeavor for both the clinician and patient. Finding the optimal treatment plan for an individual patient often involves choosing from several alternatives in a series of trial-and-error decisions. Lack of evidence regarding the relative efficacies of each treatment, limited measures to assess response, and variability of response within populations are all serious challenges. Medical conditions with this type of profile affect patients of all ages; examples include attention deficit hyperactivity disorder, chronic musculoskeletal pain, gastroesophageal reflux disease, and sleep disturbances [2–4]. Patients with chronic conditions and their providers face significant challenges in identifying an optimal treatment path that is effective and consistent with the patient’s goals of care. Pediatric populations are a specifically challenging group in that individual care coordination and distribution of responsibilities are navigated within a larger group of stakeholders, often including teachers and parents [5–7]. In this population, roles also change with age, requiring nuanced approaches to managing chronic conditions over time.

The importance of the patient as an individual is reflected in the growing focus on patient-centered care, including the medical home model [8, 9], and patient engagement as an essential dimension of improving healthcare delivery [10–12]. This approach can be further strengthened by the incorporation of patient-reported outcomes and goal setting into clinical care as a way to better align treatments with the goals of care that patients identify as most important [13–17].

These emerging trends provided the impetus for us to develop a novel treatment strategy that focuses on the individual. Currently, most electronic health record (EHR) systems do not offer functionality for systematic, patient-driven and patient-provided outcome assessments. The EHR tools that are available for capturing patient-reported outcomes are difficult to implement, rigid in terms of functionality, and limited in terms of physicians’ ability to review data from outside the EHR. Thus, these tools are challenging to incorporate into routine care for decision-making and course correction [8, 18]. In an attempt to create a solution that goes beyond these limited EHR tools, we developed a quality improvement (QI) infrastructure to support “N-of-1” treatment decision-making using patient-reported outcomes – the Vanderbilt TAILOR tool (Treatment Assessments in the Individual Leading to Optimal Regimens). The goal of our TAILOR program is to aid providers and patients in making personalized decisions by enabling systematic, detailed, and synchronous assessment of patient-reported outcomes during routine care. This report describes our retrospective review of pilot data from application of this novel patient-centered infrastructure in three pediatric-focused implementations.

## Methods

### TAILOR tool overview

**Fig. 1:**
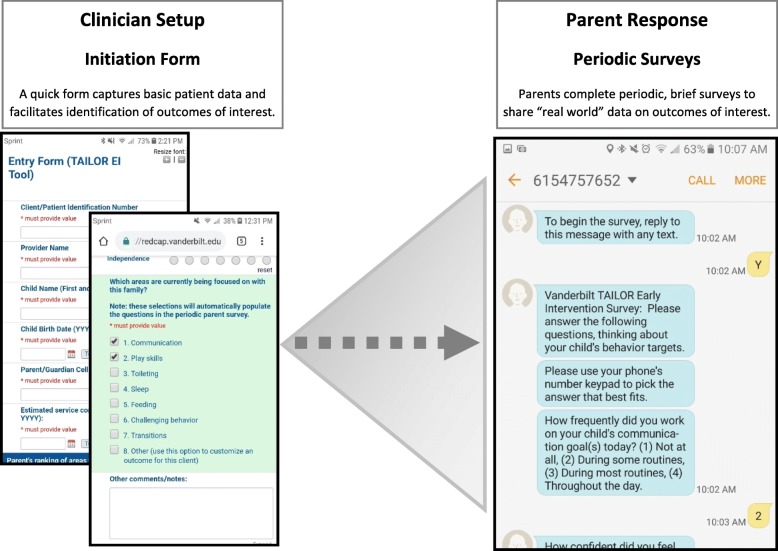
TAILOR examples: initiation/set-up form and data exchange by text messaging

**Fig. 2:**
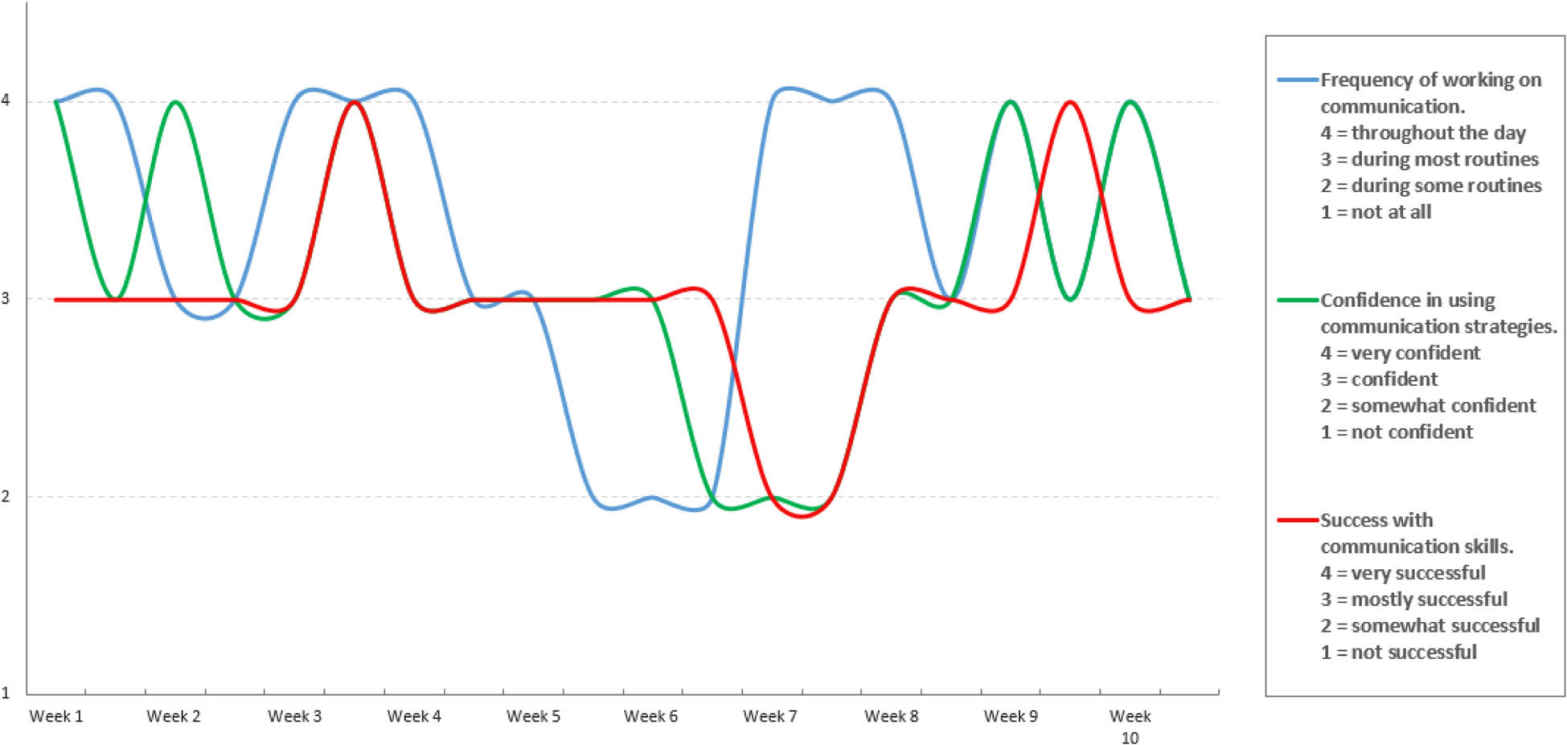
Personalized longitudinal report of patient reported outcomes in a single patient, TAILOR Early Intervention pilot project example. Caption: This figure illustrates the longitudinal data shared by an example parent during use of the TAILOR tool while receiving Early Intervention services, showing variability in data over time and enabling the provider and patient to discuss needs for refining the treatment approach. The data includes periodic snapshots of three dimensions: the frequency of work on a skill area; parent confidence in working on the skill; and child’s success in the skill area

The TAILOR tool infrastructure was developed within REDCap, a web-based software environment providing secure and customizable data collection [19]. While each TAILOR project varies somewhat based on the needs of each clinical environment, our implementations of this tool share several key features within the tool. Each TAILOR use begins with an initial set-up discussion between a team consisting of the patient (or patient’s family) and provider to select appropriate treatments to evaluate as well as patient-centered outcomes to measure. This discussion is facilitated by an intake form within TAILOR (see Fig. 1 for an example). The team also decides on frequency and timing for outcomes data collection to optimize convenience to the patient. TAILOR allows customization of these data flows by providing several different data exchange methods. Patients may share data using a mailed link to a secure web survey, by telephone, or by responding to questions via text messaging using REDCap’s integration of Twilio, a third-party text messaging service. After this set-up discussion, the treatment evaluation period begins, with periodic collection of patient-reported outcomes through the approach selected by the patient as most convenient (e.g. text messaging; see Fig. 1 for an example). Once the data collection period concludes, patient and provider are able to review outcome data (see Fig. 2 for an example report) to identify the best approach for care moving forward.

All iterations include a project-specific portal integrated into REDCap, containing a pre-programmed menu of possible outcome measures of interest and the frequency with which they are to be collected. The portal also includes supporting documentation (e.g., instructions for patients, materials for on-boarding new providers to use of the tool) to educate and inform both providers and patients. Each of the TAILOR pilots, discussed below, accessed the tool and supporting documentation (e.g., instructions for patients) using a shortened URL that was defined by the provider team.

### Pilot implementation

We implemented pilot TAILOR projects in three pediatric outpatient settings: a clinic caring for children with known or suspected attention deficit hyperactivity disorder (ADHD), a program administering early intervention (EI) services [6] to children with autism spectrum disorders or developmental delay, and a team providing support to teachers involved in the education of children with challenging behaviors (Intensive Partnership for Behavioral Intervention or IPBI). These populations were chosen due to the challenges inherent to their care management and the buy-in we were able to obtain from providers, patients, parents, and teachers. Table 1 describes each population and the approach for querying outcomes in each context, in more detail. Each pilot study iteration incorporated stakeholder perspectives and improvements in the TAILOR infrastructure. In fact, an overarching goal of these pilots was infrastructure quality improvement, aimed at continually refining the methods for facilitating collection of patient-reported outcomes and integration of these data into patient/provider decision-making.

**Table 1:** Overview of TAILOR pilot projects

Pilot population	Project description	Target population(s) Usual frequency duration of individual data collection	Launch date
Children with attention deficit hyperactivity disorder (ADHD) treated in an outpatient clinic)	The Vanderbilt ADHD TAILOR tool allowed Vanderbilt healthcare providers to send the Vanderbilt ADHD Diagnostic and/or Follow-up Rating Scale [26] questions to parents electronically. TAILOR also queried the child’s teacher with parent consent. The provider received survey results by secure email before the child’s next planned clinic visit. The ADHD pilot included one attending physician’s resident clinic.	Parent/guardian; teacher Weekly for 1–4 weeks	January 2016
Children with autism spectrum disorder or other developmental delays receiving services from the Early Intervention team	The Vanderbilt TAILOR Early Intervention (EI) tool allowed Treatment and Research Institute for Autism Spectrum Disorders (TRIAD) behavioral consultants to send brief text message surveys to a child’s parent to collect outcomes. Questions were developed based on previously employed TRIAD program assessments, and queried the frequency of work on a skill area on a given day, parent’s confidence in working on the skill, and the child’s success with a given skill. The consultant was able to view the latest survey results before each home visit.	Parent/guardian Twice/week for 3–6 weeks	April 2016
Adolescents with autism spectrum disorder and challenging behaviors receiving services from the Intensive Partnership for Behavior Intervention (IPBI) team	The TAILOR IPBI tool enabled behavioral consultants to collect information about student behavior and engagement from educators, with questions designed using existing in-house IPBI assessments. Our IPBI TAILOR implementation encompassed two separate academic years, 2016–2017 and 2017–2018.	Teacher^a^ Twice/week for 1–2 academic semesters	September 2016

Note: ^a^ a range of 1–4 teachers per child shared data using the IPBI tool

Providers in each pilot offered individuals the option to share information using the TAILOR tool as part of usual care activities. The patient’s surrogate for data collection in these pilots included parents of children affected by a chronic condition (Early Intervention Pilot, ADHD Pilot) and teachers involved in the education of a child with challenging behaviors in the school setting (IPBI Pilot). In the ADHD Pilot only, teachers also had the opportunity to use the TAILOR tool, at parents’ discretion, to share data with healthcare providers. The frequency and length of surveys were customized within each project: parents in the ADHD project received 1–5 surveys and teachers received 1–2 surveys, over a period of 1–4 weeks; in EI, parents received surveys twice weekly for the duration of EI services, typically ranging from 3 to 6 weeks; and in the IPBI project, teachers received surveys twice weekly for the duration of their participation in the program, usually 1–2 semesters of the academic year. Questions for collection of patient-reported outcomes were drawn from validated scales in one pilot (ADHD) and from previously developed in-house assessments in two pilots (EI, IPBI).

In addition to the data on patient-reported outcomes, we also collected client and provider satisfaction data in all TAILOR pilot projects using REDCap; satisfaction surveys were typically sent 1–2 weeks after completion of the pilot implementation to providers and to parents/teachers who had used TAILOR at least once to share data. We summarized utilization and satisfaction data from the TAILOR pilots using descriptive statistics (e.g., counts, percentages).

This project was reviewed and approved by our Institutional Review Board under 45.CFR 46.102 (d) as a non-research, quality improvement project. Patients were consented as part of their clinical care outside of research activities.

### Data analysis

The analysis incorporates available data from each pilot’s inception through June 2018. For each of the pilots, we extracted utilization data from REDCap, including number of participants and providers, patient outcomes of interest (EI and ADHD pilots), satisfaction data, and survey response rates. Further, as the IPBI pilot included two separate academic years, we reported utilization data for both 2016–2017 and 2017–2018.

## Results

The three pilots included a combined total of 109 children and 39 healthcare providers, with 67 parents and 77 teachers invited to share data using the TAILOR infrastructure (Table 2). In the ADHD project, the majority of parents elected to include a teacher in the data collection process, with 22 teachers receiving a diagnostic and/or treatment follow-up assessment via the TAILOR tool. In the two pilots providing the option for data collection by text messaging, this mode of data collection predominated in the EI population (93%), while a relatively smaller proportion used text messaging in the IPBI cohorts (31–50%) as compared with email survey invitations.

**Table 2:** TAILOR utilization characteristics

Pilot area	Children (n)^a^	Parents (n)	Teachers (n)	Providers (n)	Participants preferring to complete surveys by email link (%)	Participants preferring to complete surveys by text messaging (%)	Response rate, completed surveys/surveys sent (%)
ADHD	23^b^	23	22	18	100%	NA^c^	Parents: 30/79 (38%) Teachers: 24/29 (83%)
EI	44	44	NA	8	7%	93%	209/443 (47%)
IPBI 2016–2017	19	NA	29	6	69%	31%	611/725 (84%)
IPBI 2017–2018	23	NA	26	7	50%	50%	932/1178 (79%)

Note: ^a^ Represents the number of children with a parent (ADHD, EI) or teacher (IPBI) using TAILOR to share data with a provider

^b^Represents unique records; 5 parents used the tool more than once

^c^The ADHD TAILOR tool did not include the option to complete surveys by text messaging

Two of the tools (ADHD, EI) allowed for personalized selection of outcomes for periodic monitoring during treatment, while the IPBI tool collected standard outcome data for all participants. Among the 18 parents in the ADHD pilot who selected personalized outcomes for periodic monitoring, the three most-selected outcomes included classroom behavior (*n* = 13, 72%), sustaining attention to tasks/activities (*n* = 11, 61%), and completing assignments (*n* = 9, 50%). The three most common parent-selected areas tracked in the EI TAILOR project were communication (*n* = 25, 57%), challenging behavior (*n* = 15, 34%), and play skills (*n* = 6, 14%).

Overall survey response rates ranged from 38% to 84% across the three pilots. Response rates among parents ranged from 38% to 47% (ADHD and EI, respectively), while response rates for teachers varied from 79% to 84% among the different pilots in which they were included (ADHD, IPBI).

A small number of tool users answered a brief TAILOR satisfaction survey, including 12 patients/surrogates and 19 providers, representing a response rate of 11% and 49%, respectively. Responses from both user groups indicated widespread satisfaction measured across all domains, with most respondents agreeing or strongly agreeing with all statements about the tool’s utility (Table 3). Notably, the improvement in provider-patient communication endorsed by both user groups (100% and 94%) was a compelling result, reinforcing our belief that a major benefit of the tool’s usage would be an increase in informed collaborative decision-making.

**Table 3:** Patient/surrogate and provider assessment of satisfaction with the TAILOR tool

Aggregate satisfaction domains across pilots	Total responses, n	Agree or strongly agree rating, n (%)
Patient/surrogate (parent, teacher)		
Data improved communication with provider	12	12 (100)
TAILOR was a convenient way to share information with provider	12	11 (91.7)
Helped understand outcomes of interest	12	12 (100)
Helped provider make best plan for child/student care	12	12 (100)
Helped provider understand client challenges and successes of implementing intervention.	12	11 (91.7)
Helped patient/surrogate understand the successes of intervention	12	11 (91.7)
Gave information that helped patient/surrogate and provider make decisions about child/student care	12	12 (100)
TAILOR tool helped patient/surrogate track outcomes of interest ^a^	7	6 (85.7)
Would recommend TAILOR tool to others ^a^	7	6 (85.7)
Felt more involved in decisions about child/student care ^b^	2	2 (100)
Interested in using TAILOR tool to support decision making for child/student care in the future ^b^	2	2 (100)
Providers (healthcare provider, consultant)		
Improved communication with patient/surrogate	18	17 (94.4)
Increased patient/surrogate engagement in decision making	18	17 (94.4)
Helped provider understand efficacy of interventions for child/student	18	18 (100)
Gave provider information that helped decision making and communication with patient/surrogate about child/student care	18	16 (88.9)
TAILOR tool was a convenient way to collect information from patient/surrogate	18	18 (100)
Using the TAILOR tool took a reasonable amount of time	19	19 (100)
The periodic survey data helped provider better understand trends in child/student outcomes of interest	18	16 (88.9)
Would recommend TAILOR tool to others	19	18 (94.7)
Reviewing the survey data took a reasonable amount of time ^d^	15	14 (93.3)
Gathered useful data from the child/student’s teacher ^b^	2	2 (100)
Would be interested in using a tool like TAILOR in the future^b^	4	3 (75)
Helped provider identify needs with patient/surrogate for future support and intervention ^c^	3	2 (66.7)

Key: ^a^ asked in ADHD and IPBI pilots only; ^b^ asked in ADHD pilot only; ^c^ asked in IPBI pilot only; ^d^ asked in ADHD and EI pilots only

## Discussion

These three pilot studies demonstrated the TAILOR tool’s utility in collecting patient-reported outcomes from the “real world” while a patient tries a course of therapy. Moreover, the TAILOR tool proved valuable for facilitating communication between parents, teachers and providers regarding each patient’s symptoms and goals for treatment.

The tool also showed potential value in its method of gathering data to form a more holistic view of the patient. Not only did satisfaction data indicate the overall value of the data for helping understand the child, but the tool also proved effective in engaging the teacher, a factor significantly associated with positive outcomes in children with ADHD or autism spectrum disorders [20–22]. In the ADHD project, for example, teachers were regularly included in the TAILOR process by parents and frequently completed the requested outcome surveys, interestingly at a rate higher than the parents themselves. The providers in the ADHD pilot further noted anecdotally that the number of TAILOR-generated forms they received from teachers was much higher as compared to what they generally receive using a traditional process (e.g., giving the parent a form to have the teacher complete and return to the parent, or faxing the form to the child’s school). These findings again suggest the tool’s utility as a complement to usual practices. Teachers using the TAILOR tool for IPBI were similarly engaged in the data collection process with respect to children receiving intervention and support from IPBI consultants. In this case, teachers shared periodic written snapshots of classroom experiences that provided consultants with opportunities to further support the children and their teachers. Thus, our pilot experiences suggest that the TAILOR tool may be a useful strategy for adding dimensionality to patient data by facilitating information collection that reflects more than one setting and stakeholder perspective for a child.

Variability in user messaging preferences suggests that customization of the data flow experience may be an important part of future iterations. In the EI pilot, for example, parents almost exclusively preferred text messages, while teacher preferences in the IPBI pilots were distributed more evenly between email (often at their work address) or text messaging (to their personal cell phone). Future work will help us learn more about which areas of customization are most needed to ensure convenience and completeness of data collection among different populations.

The limitations of this research suggest implications for future research and clinical implementation. For example, we did not collect demographic data, and thus are not able to comment on any potential variation among possible subgroups. Also, while we observed relatively high response rates to outcome surveys among teachers, response rates were notably lower among parents, indicating that user engagement could be improved. Additional work is needed to explore ways to increase this engagement. Response rates to our satisfaction survey were low, suggesting that gathering additional formative data to further optimize the TAILOR infrastructure could be of value.

### Future directions

While this report demonstrates the value of our “N-of-1” approach for use in everyday clinical practice, we believe it will prove equally beneficial in a research application. We are currently leveraging our successful pilot experiences with the TAILOR infrastructure to support development and implementation of an N-of-1 randomized controlled trial (RCT) [23] to evaluate comparative effectiveness of interventions in a chronic pain clinic population, with anticipated study start in 2019. As a part of this research process, we are also developing additional methods that will facilitate generalizability of this approach to future studies, including statistical methods for analyzing data within the individual patient as well as meta-analytic approaches for analyzing data across patients within the N-of-1 RCT. A chronic pain study will likely have different implementation needs than the pediatric pilot studies we describe above; we hope that learning how to generalize this approach to a new population will further our ability to export this technique for other uses and for use by any interested investigator. Thus, after adapting and refining TAILOR further, we hope to disseminate this infrastructure to facilitate N-of-1 studies and the collection of patient-reported outcomes. Our future aim is to make N-of-1 research protocols easy to implement within a range of clinical settings and to further strengthen our collective capacity to explore personalized medicine hypotheses.

## Conclusions

These pilot experiences show the utility of the TAILOR approach for supporting collection and incorporation of patient-reported outcomes into the care of individuals with chronic conditions. The growing recognition of the importance of patient-reported outcomes and emergence of technologies to support convenient data collection provide excellent support for the advancement of N-of-1 methods for personalized patient care [24, 25].

## Abbreviations

ADHD: Attention deficit hyperactivity disorder
EHR: Electronic health record
EI: Early Intervention
IPBI: Intensive Partnership for Behavioral Intervention
REDCap: Research electronic data capture
TAILOR: Treatment Assessments in the Individual Leading to Optimal Regimens
